# Enhanced absorption of prenylated cinnamic acid derivatives from Brazilian green propolis by turmeric in humans and rats

**DOI:** 10.1002/fsn3.4116

**Published:** 2024-04-04

**Authors:** Masayuki Yamaga, Hiroshi Kawabe, Hiroko Tani, Ayanori Yamaki

**Affiliations:** ^1^ Institute for Bee Products and Health Science, Yamada Bee Company, Inc. Tamata‐gun Okayama Japan

**Keywords:** artepillin C, Brazilian green propolis, breast cancer resistant protein, cinnamic acid derivatives, drupanin, turmeric

## Abstract

Prenylated cinnamic acid derivatives are the bioactive components of Brazilian green propolis (BGP). The effect of other botanical components on the pharmacokinetic profiles of these derivatives remains relatively unexplored. In the present study, we investigated the influence of several herbal extracts (turmeric, ginkgo leaf, coffee fruit, soybean, and gotu kola) on the plasma concentrations of cinnamic acid derivatives after BGP consumption. When the herbal extracts were co‐administered with BGP in the clinical study, the area under the curve (AUC) values of artepillin C and drupanin, the major BGP components in plasma, were significantly increased by 1.7‐ and 1.5‐fold, respectively, compared to those after BGP administration alone. Among the herbal extracts administered to rats, turmeric extract increased the AUC. Furthermore, a bidirectional transport assay suggested that artepillin C and drupanin are substrates of breast cancer resistance protein (BCRP), a drug elimination transporter. These results suggest that curcumin‐containing turmeric extract may increase the plasma concentrations of artepillin C and drupanin via BCRP. Our findings enabled us to estimate the food–herb and herb–herb interactions in vivo in foods and herbal medicines containing cinnamic acid derivatives and prenylated compounds.

## Graphical abstract

This study investigated the effects of several herbal extracts (turmeric, ginkgo leaf, coffee fruit, soybean, and gotu kola) on the plasma concentrations of cinnamic acid derivatives after Brazilian green propolis (BGP) ingestion. In the clinical study, the AUC of artepillin C, a major plasma component of BGP, was significantly increased by 1.7‐fold after co‐administration of the herbal extracts and BGP compared with the administration of BGP alone. Among the herbal extracts, turmeric increased the AUC of artepillin C in rats.

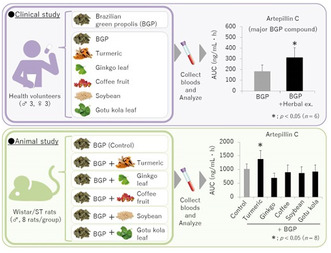

## 1 INTRODUCTION

Propolis is a resinous material produced by honeybees (*Apis mellifera* L.) from the leaf buds and barks of various plant species (Bankova et al., 2000; Burdock, 1998; Ghisalberti, 1979). The botanical origin and composition of propolis vary according to the vegetation in the production area (Toreti et al., 2013). The botanical origin of Brazilian green propolis (BGP), produced in the state of Minas Gerais in southeastern Brazil, is *Baccharis dracunculifolia* (Park et al., 2002). BGP contains characteristic prenylated cinnamic acid derivatives, such as artepillin C, drupanin, capillartemisin A, and 3,4‐dihydroxy‐5‐prenylcinnamic acid (Tani et al., 2019), which have been reported to inhibit cys‐leukotriene release (Tani et al., 2010), induce beige adipocyte formation in white adipose tissue (Nishikawa et al., 2020), inhibit PAK1 signaling (Takahashi et al., 2017), and induce neurite outgrowth (Kano et al., 2008). Therefore, these derivatives are expected to be the active components responsible for the various effects of BGP, such as antitumor (Bhargava et al., 2018), anti‐inflammatory effects (Wu et al., 2013), mitigation of cold symptoms and allergic rhinitis (Cardoso et al., 2016; Fiordalisi et al., 2016), improvement of obesity (Koya‐Miyata et al., 2009), and alleviation of SARS‐Cov‐2 (Silveira et al., 2021). Recently, Zhu et al. (2018) and Asama et al. (2021) reported improved cognitive function in elderly people owing to the long‐term intake of BGP, with artepillin C suggested as the potential active component. A mixture of BGP and several herbal extracts (turmeric, ginkgo leaf, coffee fruit, soybean, and gotu kola) has been found to improve cognitive function in the elderly (Asama et al., 2020). Curcumin in turmeric, ginkgolides in ginkgo leaves, phosphatidylserine from soybeans, and chlorogenic acid in coffee fruits have been reported to reduce neuroinflammation and exert neuroprotective effects (De Simone et al., 2003; Liu et al., 2023; Xiong et al., 2023; Yu et al., 2018). Additionally, gotu kola presumably improves memory in humans (Fitriana et al., 2021). Hence, BGP and these herbal extracts may have a synergistic effect on cognitive function.

Generally, the biological activities and pharmacokinetics of bioactive constituents are influenced by the presence of other constituents in food and herbal plants (Phan et al., 2018; Scholz & Williamson, 2007). Several studies have revealed enhanced absorption of herbal constituents due to the co‐administration of several herbs (Sun et al., 2019). Recently, green propolis has been reported in vitro to improve the intestinal membrane permeability of curcuminoids (de Albuquerque et al., 2023), suggesting interaction between components of natural products, thereby affecting their absorption. Therefore, the absorption of BGP constituents is affected by their interaction with other herbs. In our previous studies, we evaluated the uptake and metabolic processes of cinnamic acid derivatives of BGP in humans and rats (Yamaga et al., 2021, 2022). In particular, artepillin C and drupanin were the primary constituents detected in the plasma after BGP consumption, and some of the components were metabolized to conjugates and hydroxylated metabolites, such as capillartemisin A and 3,4‐dihydroxy‐5‐prenylcinnamic acid. Baccharin was hydrolyzed to drupanin and 3‐phenylpropionic acid and was not detected in any plasma sample. Notably, few studies have investigated the phytochemicals that affect the absorption of cinnamic acid derivatives. Therefore, the objective of this study was to determine the effects of herbal extracts (turmeric, ginkgo leaf, coffee fruit, soybean, and gotu kola) on the absorption of cinnamic acid derivatives of BGP. Herein, we describe the effect of the interaction between BGP and herbal extracts on the pharmacokinetic profiles of cinnamic acid derivatives after a mixture of BGP and herbal extracts was consumed by humans, and a combination of BGP and each herbal extract was ingested by rats. In addition, we discuss the molecular mechanisms underlying this interaction using a cell‐based bidirectional transport assay.

## 2 MATERIALS AND METHODS

### 2.1 Materials and chemicals

BGP and natural herbal extracts (turmeric, ginkgo leaf, coffee fruit, soybean, and gotu kola) were obtained as freeze‐dried powders from Yamada Bee Company, Inc. (Okayama, Japan). The BGP powder (Lot: LY‐008) was standardized to contain 8.0% artepillin C and 0.14% culifolin. Turmeric extract powder was standardized to contain 70% curcumin, 15% desmethoxycurcumin, and 2.5% *bis*‐demethoxycurcumin. Gingko leaf extract powder was standardized to contain 24% total flavonol glycosides and 6% total terpene lactones. Coffee fruit extract powder and soybean extract powder were standardized to contain 40% chlorogenic acid and 70% phosphatidylserine, respectively. For the clinical trial, the extract powders were mixed and encapsulated according to the proportions mentioned previously (Asama et al., 2020): 75 mg of BGP powder; 250 mg of turmeric extract powder; 120 mg of ginkgo leaf extract powder; 100 mg of coffee fruit extract powder; 150 mg of soybean extract powder; and 250 mg of gotu kola extract powder. Authentic artepillin C was procured from Fujifilm Wako Pure Chemical Industries Ltd. (Osaka, Japan). Drupanin, capillartemisin A, and 3,4‐dihydroxy‐5‐prenylcinnamic acid were prepared according to the methods outlined in previous reports (Tani et al., 2010, 2019). Sulfatase from *Helix pomatia* (type H‐1 with a minimum activity of 10,000 units/g solid, including β‐glucuronidase with a minimum activity of 300 units/mg solid) was purchased from Sigma‐Aldrich Inc. (St. Louis, MO, USA). All reagents were of analytical grade.

### 2.2 In vitro study

The Madin‐Darby canine kidney (MDCK II) cell line and MDCK II cells overexpressing multidrug resistance 1 (MDR1) or breast cancer resistance protein (BCRP) were purchased from the Netherlands Cancer Institute (Amsterdam, Netherlands) and SOLVO Biotechnology (Szeged, Hungary), respectively. The cell lines were routinely cultured in Dulbecco's modified Eagle's medium (DMEM) containing 10% fetal bovine serum and 1% penicillin/streptomycin at 37°C in 5% CO_2_. All cells were seeded in a feeder tray at a density of 3–6 × 10^4^ cells/well and grown for 3–4 days. On the day of the transport study, cell monolayers were washed with Hanks' balanced salt solution (HBSS) buffer containing 0.1% BSA (pH 7.4) and incubated at 37°C for 20 min. Artepillin C and drupanin dissolved in DMSO (10 mM) were added to the transport buffer (HBSS containing 10 mM HEPES, 0.2% glucose, 0.1% BSA, pH 7.4) to a final concentration of 10 μM and then loaded into the apical or basolateral chamber. After 120 min of incubation, 150 μL of the aliquots were collected from the donor and receiver chambers and mixed with an equal volume of 50% methanol. After centrifugation at 3000 rpm for 10 min at 4°C, the supernatants were filtered and subjected to LC–MS/MS analysis.

### 2.3 Animal study

This animal study was approved by the Animal Research Ethics Committee of KAC Co., Ltd. (Kyoto, Japan; approval No. 22‐1217). All rats were handled in accordance with the Animal Care and Use Regulations of KAC Co., Ltd.

Wistar/ST rats (male, 7 weeks old) were purchased from Japan SLC (Shizuoka, Japan). Forty‐eight rats were housed in laminar flow cages under the following conditions: temperature 23 ± 2°C; relative humidity 45%–60%; 12 h light/dark cycle (from 7:00 a.m. to 7:00 p.m.); and with ad libitum access to water and food. The rats were quarantined and acclimatized under the above conditions for 1 week and divided into six groups (eight rats/group) according to their average body weight (240–260 g). Each test sample listed in Table 1 was dissolved in 10% propylene glycol (1 mL per rat) and administered intragastrically to the rats after an overnight fasting period with free access to water. The dosage ratios of the test samples were determined based on the proportions shown to improve cognitive function in a previous study (Asama et al., 2020). Blood samples were obtained from the tail vein of rats using heparin‐coated glass capillaries (Thermo Scientific, Waltham, MA, USA) before the test component administration as well as at intervals of 30 min and 1, 1.5, 2, 3, 6, 9, and 24 h after the administration. The collected samples were centrifuged at 1500 *g* for 10 min at 4°C to separate the plasma and preserved at −80°C until further sample preparation.

**TABLE 1:** Combination and dosage of Brazilian green propolis (BGP) and each herbal extract administered to rats.

Group	Ingestion samples (mg/kg)					
	BGP	Turmeric	Ginkgo leaf	Coffee fruit	Soybean	Gotu kola
1	75	–	–	–	–	–
2	75	250	–	–	–	–
3	75	–	120	–	–	–
4	75	–	–	100	–	–
5	75	–	–	–	150	–
6	75	–	–	–	–	250

### 2.4 Clinical trial

The protocol for the clinical trial was reviewed and approved by the Yamada Bee Co. Ethical Committee. This study adhered to the ethical tenets of the Declaration of Helsinki (approval no. 2020015). All participants were informed of the research protocol, and they have signed an informed consent form.

Six volunteers (three males and three females) completely understood the study protocol and consented to participate in the research. The following exclusion criteria were used for shortlisting participants: past or present medical history (asthma, significant allergies, heart, liver, kidney, and urinary tract disease); prescribed medication; pregnancy; smoking (>20 cigarettes/day); alcohol consumption (>500 mL/day of beer); use of dietary supplements and healthy foods; and participation in other clinical trials. The participants were instructed to refrain from consuming propolis and propolis‐containing foods and beverages during the study period and 1 week prior to the start of the study. After a 12‐h fast, the participants were given a test food comprising the encapsulated BGP powder or a mixture of the BGP powder and herbal extracts in two experimental periods separated by 1 week. Blood samples were collected before the ingestion of test food and at 30 min and 1, 1.5, 3, 6, 9, and 24 h after the ingestion. The collected samples were centrifuged at 1500 *g* for 3 min at 23 ± 2°C to separate the plasma, which was subsequently preserved at −80°C until further sample preparation.

### 2.5 Plasma sample preparation

Human and rat plasma samples were prepared as previously described (Yamaga et al., 2021, 2022). Briefly, 50 μL of plasma was treated with a sulfatase/β‐glucuronidase solution (sulfatase 15 units/sample) in 100 mM sodium acetate and 5 mM ascorbate at 37°C for 1 h. Following the incubation, 850 μL of methanol was added to the samples to stop the deconjugation reaction, after which centrifugation was performed at 2000  *g* for 10 min at 23 ± 2°C. The supernatant was evaporated under nitrogen gas and then reconstituted in a fixed volume of methanol. The samples were filtered through a 0.22 μm PTFE membrane and then analyzed by LC–MS/MS.

### 2.6 LC–MS/MS analysis

To quantify cinnamic acid derivatives in the plasma, LC–MS/MS analysis was performed using a UHPLC system (Ultimate 3000, Thermo Scientific) coupled to an Orbitrap MS system (Q‐Exactive Focus, Thermo Scientific) according to a previously described method (Yamaga et al., 2021, 2022). Briefly, 5 μL of the sample was injected into a reversed‐phase column (Acquity UPLC BEH C18, 2.1 × 100 mm, ID. 1.7 μm; Waters, Milford, MA, USA) held at 30°C. The elution was performed using solvent A (0.1% formic acid) and solvent B (acetonitrile) at a flow rate of 0.3 mL/min. The gradient profiles were as follows: 5% B (0–2 min), 5–95% B (2–22 min), 95% B (22–25 min), and 5% B (25–30 min). Tandem mass spectrometry (MS/MS) was conducted using an ion trap mass spectrometer with a heated electrospray ion source (HESI) in both positive and negative ionization modes (scan range *m/z* 70–1000). The mass detection parameters were the same as previously described (Tani et al., 2023). External standard curves for cinnamic acid derivatives were generated using eight concentrations of authentic standard solutions (1, 5, 10, 50, 100, 250, 500, and 1000 ng/mL).

The in vitro samples were analyzed using HPLC (LC‐20A, Shimadzu, Kyoto, Japan) with a reversed‐phase column (Inertsil ODS‐3, 2.1 × 33 mm, ID. 3 μm, GL Sciences, Tokyo, Japan) held at 40°C. The mobile solvents used were water with 10 mM ammonium acetate (pH 4.0, A) and methanol (B) pumped at a flow rate of 0.4 mL/min. The following gradient was employed: 10%–90% B (0–0.5 min), 90% B (0.5–2.5 min), and 10% B (2.5–4 min). The eluent was monitored using a triple quadrupole mass spectrometer (API 4000, AB Sciex, Framingham, MA, USA) incorporating an electrospray ion source (ESI) operating in the multiple reaction monitoring mode. The ESI source was operated in negative ionization mode at 600°C with a spray voltage of −4.5 kV. The curtain gas (nitrogen), collision gas (nitrogen), nebulizer gas (whole air), and drying gas (whole air) were set at 40, 5, 50, and 70 psi, respectively. The monitor ions of artepillin C and drupanin were *m/z* 299/255 [M − H]^−^ and *m/z* 231/132 [M − H]^−^, respectively. Artepillin C and drupanin were diluted in 50% methanol to concentrations of 1.6, 3.2, 8, 40, 200, 1000, and 2000 nM and were used as external standards. Sulfaphenazole (0.2 μM) was used as an internal standard.

### 2.7 Pharmacokinetic analysis

Quantification of cinnamic acid derivatives in the plasma was performed using the Trace Finder software version 4.1 (Thermo Scientific). The plasma concentration versus time data for the analytes obtained from each human and rat were entered into a Microsoft Excel spreadsheet. The area under the plasma concentration–time curve (AUC) was calculated using the linear trapezoidal rule, extending to the last sampling point.

### 2.8 Data analysis

Plasma concentration data were expressed as mean ± standard deviation, and statistical analyses were performed using GraphPad Prism 7 (GraphPad Software, San Diego, CA, USA). In the clinical study, differences in the plasma concentrations and AUC values of the cinnamic acid derivatives due to the intake of the test foods were analyzed using the paired *t*‐test. For the animal study, two‐group comparisons were carried out using Student's *t*‐test, while multiple comparisons were conducted using one‐way analysis of variance (ANOVA) followed by Dunnett's post hoc test. Significant differences in the measured variables were recognized when the derived *p*‐value was ≤.05.

The apparent permeability coefficients (Papp) for both directions, from the apical side to the basolateral side (A to B) and from the basolateral side to the apical side (B to A) in the cell monolayer, were calculated using the following equation:

(1) $$\text{Papp}=\left(\mathrm{dQ}/\mathrm{dt}\right)/\left(A\times \text{C0}\right)$$

where dQ/dt is the permeation rate (nmol/s), A is the membrane area (cm^2^), and C0 is the initial concentration of the compound in the donor compartment (nM). The efflux ratios (ER) in the B‐to‐A and A‐to‐B directions were determined using the following equation:

(2) $$\mathrm{ER}=\text{Papp}\;\left(B\;\text{to}\;A\right)/\text{Papp}\;\left(A\;\text{to}\;B\right)$$

Net flux ratios were calculated by dividing the ER obtained from MDCK II‐MDR1 (or BCRP) cells by that obtained from the parent MDCK II cells. A net flux ratio greater than 2 indicated that the efflux of the compound was specifically mediated by the transporter (Huang et al., 2010).

## 3 RESULTS

### 3.1 Co‐administration of a mixture of herbal extracts increased the absorption of BGP‐derived cinnamic acid derivatives in humans

**FIGURE 1:**
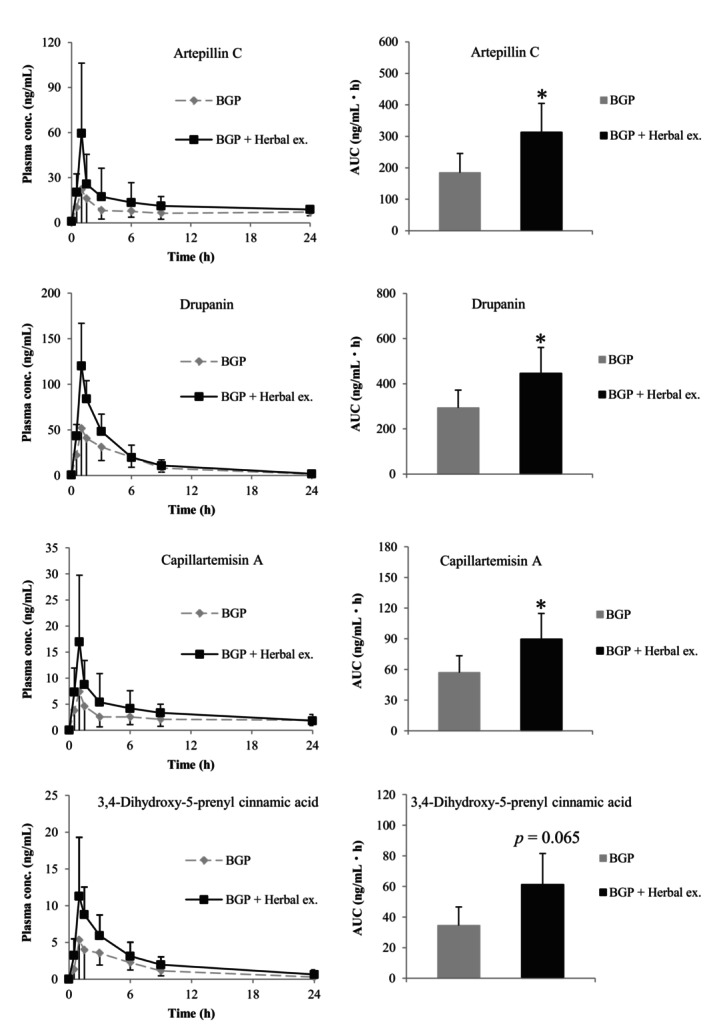
Plasma concentration–time curve and area under the curve (AUC) of the prenylated cinnamic acid derivatives in humans (mean ± SD; *n* = 6) within 0–24 h after ingestion of Brazilian green propolis (BGP) alone or a mixture of BGP and the herbal extracts. The plasma concentrations were analyzed using LC–MS/MS after enzymatic deconjugation. The AUC was calculated using the trapezoidal rule. *p < .05.

To determine the effect of the herbal extract mixture on the absorption of the cinnamic acid derivatives of BGP in humans, plasma samples were collected for up to 24 h from participants who received BGP capsules or capsules containing a mixture of BGP and herbal extract powder. After enzymatic deconjugation, the plasma concentrations of artepillin C, drupanin, and their hydroxylated forms, capillartemisin A and 3,4‐dihydroxy‐5‐prenylcinnamic acid, were analyzed by LC–MS/MS. Figure 1 shows the plasma concentration–time curves and AUC of these components. When BGP and the herbal extracts were co‐administered, the AUC values of artepillin C and drupanin were 312.9 ± 91.4 and 446.2 ± 144.5 ng/mL h, respectively; these values were 1.7‐ and 1.5‐fold higher than those obtained when BGP was administered alone (*p* < .05 for each). Furthermore, when BGP and the herbal extracts were co‐administered, the AUC values of capillartemisin A and 3,4‐dihydroxy‐5‐prenylcinnamic acid were 57.0 ± 16.5 and 34.5 ± 12.2 ng/mL h, respectively; these values were 1.6‐ and 1.8‐fold higher than those obtained when BGP was administered alone (*p* < .05, *p* = .065, respectively). These results indicate that some components of the herbal extract mixture contribute to the increased absorption of the cinnamic acid derivatives of BGP.

### 3.2 Turmeric extract enhanced the absorption of the cinnamic acid derivatives of BGP

**FIGURE 2:**
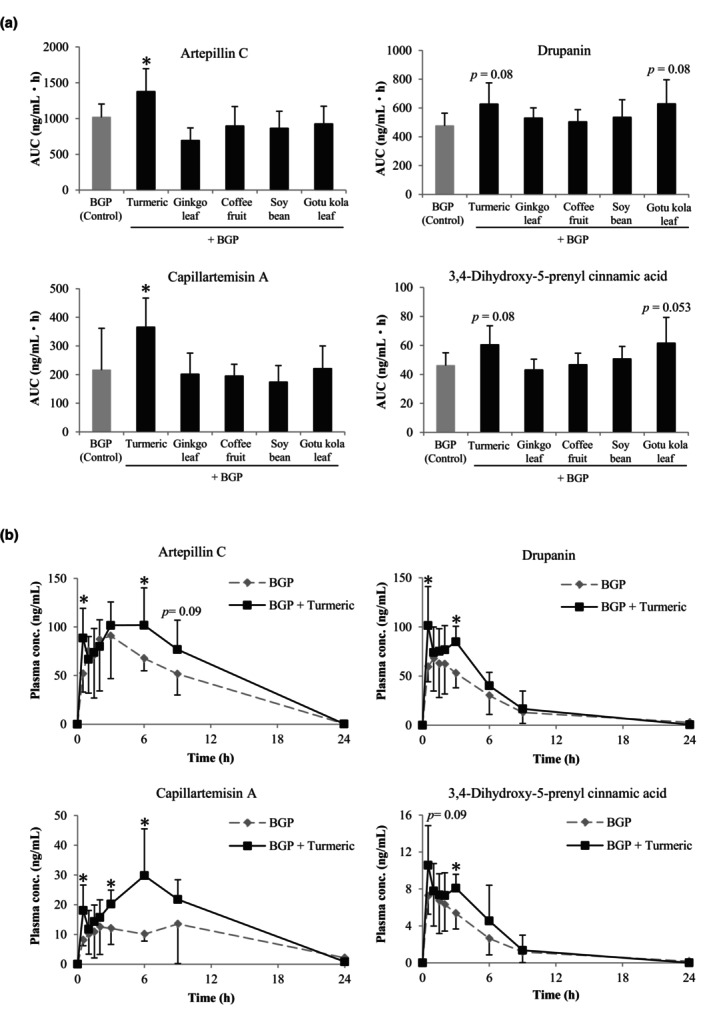
(a) Area under the curve (AUC) of the prenylated cinnamic acid derivatives when Brazilian green propolis (BGP) was co‐administered with each herbal extract. The AUC was calculated using the trapezoidal rule. (b) Comparison of the plasma concentration–time curve of prenylated cinnamic acid derivatives in the BGP group (control) and the BGP + turmeric group. All data are expressed as mean ± SD, *n* = eight rats. *p < .05 versus BGP (control).

To identify the herbal extracts that affect the absorption of cinnamic acid derivatives of BGP, rats were co‐administered BGP and each herbal extract (turmeric, ginkgo leaf, coffee fruit, soybean, and gotu kola). Plasma samples were collected for up to 24 h after the ingestion of each mixture and subjected to LC–MS/MS analysis following enzymatic deconjugation. Figure 2a shows the AUC values for artepillin C, drupanin, capillartemisin A, and 3,4‐dihydroxy‐5‐prenylcinnamic acid when each herbal extract was co‐administered with BGP.

When BGP was co‐administered with turmeric extract, the AUC value of artepillin C was 1377.2 ± 320.2 ng/mL h, which was significantly 1.4‐fold higher than that when BGP was administered alone (1018.0 ± 184.5 ng/mL h, *p* < .05). In contrast, the co‐administration of ginkgo leaf, coffee fruit, soybean, or gotu kola with BGP did not significantly alter the AUC values of artepillin C. When BGP was co‐administered with turmeric extract, the AUC value of capillartemisin A was 365.7 ± 184.5 ng/mL h, a significant 1.7‐fold increase compared with that obtained when BGP was administered alone (*p* < .05). Although no significant difference was found for drupanin when turmeric or gotu kola extract was co‐administered with BGP, the AUC values were 626.9 ± 147.7 and 629.2 ± 166.8 ng/mL h, respectively. These values tended to increase 1.3‐fold compared with those obtained when BGP was administered alone (*p* = .08 each). The AUC values also tended to increase with 3,4‐dihydroxy‐5‐prenylcinnamic acid (*p* = .08 and .053, respectively).

The plasma concentration–time curves of cinnamic acid derivatives after the co‐administration of BGP and turmeric extract were compared with those after the administration of BGP alone (Figure 2b). The plasma concentration–time curve of artepillin C exhibited two distinct peaks at 30 min and 6 h after the co‐administration of BGP and turmeric extract, and the concentrations were significantly increased by 1.7‐ and 1.5‐fold, respectively, compared to those after the administration of BGP alone (*p* < .05). Furthermore, the plasma concentrations of capillartemisin A at 30 min, 3 h, and 6 h after the co‐administration of BGP and turmeric extract were 2.2‐, 1.7‐, and 3.0‐fold higher than those after BGP ingestion, respectively (*p* < .05, for each). The plasma concentration–time curves of drupanin and 3,4‐dihydroxy‐5‐prenylcinnamic acid also revealed significant increases or increasing trends at 30 min and 3 h after the co‐administration of BGP and turmeric extract compared to the ingestion of BGP alone. These results indicate that among the herbal extracts, turmeric extract increased the absorption of cinnamic acid derivatives in BGP.

### 3.3 Cinnamic acid derivatives derived from BGP could be substrates for the drug elimination transporters

To determine whether artepillin C and drupanin are substrates for the drug elimination transporters P‐gp and BCRP, their net flux ratios were calculated using an MDCK II cell monolayer overexpressing each transporter. Tables 2 and 3 show the results of the permeability studies using artepillin C and drupanin. The Papp (A to B) and Papp (B to A) values of artepillin C in MDCK II cells were 14.0 ± 0.7 and 11.2 ± 1.2 × 10^−6^ cm/s, respectively, and the ER value was 0.8. In MDCK II‐MDR1 cells overexpressing P‐gp, the ER value of artepillin C was 0.9 and did not change with P‐gp overexpression. Similarly, the ER values of drupanin in the MDCK II and MDCK II‐MDR1 cell monolayers were 0.9 and 0.8, respectively (Table 2).

**TABLE 2:** Bidirectional Papp, efflux ratio, and net flux ratio of artepillin C and drupanin (10 μM) across MDCK II and MDCK‐MDR1 cell monolayers.

	Papp MDCK II A to B (10^−6^ cm/s)	Papp MDCK II B to A (10^−6^ cm/s)	Papp MDCK II‐MDR1 A to B (10^−6^ cm/s)	Papp MDCK II‐MDR1 B to A (10^−6^ cm/s)	Flux ratio (MDCK II)	Flux ratio (MDCK II‐MDR1)	Net flux ratio
Artepillin C	14.0 ± 0.7	11.2 ± 1.2	13.3 ± 0.7	11.4 ± 0.8	0.8	0.9	1.1
Drupanin	11.1 ± 0.2	9.9 ± 0.2	13.3 ± 1.4	11.2 ± 0.2	0.9	0.8	0.9
Parazosin^a^	19.7 ± 0.5	18.3 ± 0.3	10.3 ± 0.9	22.8 ± 0.2	0.9	2.2	2.4

*Note*: Mean ± SE (*n* = 2).

Abbreviation: Papp, Apparent permeability coefficient.

^a^ Positive control.

**TABLE 3:** Bidirectional Papp, efflux ratio, and net flux ratio of artepillin C and drupanin (10 μM) across MDCK II and MDCK‐BCRP cell monolayers.

	Papp MDCK II A to B (10^−6^ cm/s)	Papp MDCK II B to A (10^−6^ cm/s)	Papp MDCK II‐BCRP A to B (10^−6^ cm/s)	Papp MDCK II‐BCRP B to A(10^−6^ cm/s)	Flux ratio (MDCK II)	Flux ratio (MDCK II‐BCRP)	Net flux ratio
Artepillin C	13.0 ± 0.8	10.8 ± 0.1	9.7 ± 0.2	16.0 ± 0.01	0.8	1.6	2.0
Drupanin	11.6 ± 0.1	8.9 ± 0.4	1.3 ± 0.1	20.9 ± 0.1	0.8	15.7	20.4
Parazosin^a^	17.4 ± 0.5	21.4 ± 0.4	1.0 ± 0.1	41.1 ± 0.9	1.2	42.3	34.4

*Note*: Mean ± SE (*n* = 2).

Abbreviation: Papp, Apparent permeability coefficient.

^a^ Positive control.

In the MDCK II‐BCRP cell monolayer, the Papp (A to B) and Papp (B to A) values of artepillin C were 9.7 ± 0.2 and 16.0 ± 0.01 × 10^−6^ cm/s, respectively, and those of drupanin were 1.3 ± 0.1 and 20.9 ± 0.1 × 10^−6^ cm/s, respectively. The ER values of artepillin C and drupanin in cells overexpressing BCRP were 1.6 and 15.7, respectively, whereas those in the parental cell line were 0.8 each. The net flux ratios of artepillin C and drupanin were 2.0 and 20.4, respectively, suggesting that BCRP mediated the efflux of artepillin C and drupanin as substrates (Table 3).

## 4 DISCUSSION

In this study, the co‐administration of turmeric extract with BGP increased the absorption of prenylated cinnamic acid derivatives of BGP. Moreover, artepillin C and drupanin, the main cinnamic acid derivatives of BGP, were confirmed to be substrates of the drug elimination transporter, BCRP.

The turmeric extract was standardized to contain 70% or more curcumin. Curcumin has been reported to inhibit P‐gp and BCRP, which are drug elimination transporters (Lee et al., 2011; Zou et al., 2020), and affect the absorption and excretion of several compounds that act as P‐gp and/or BCRP substrates (Ge et al., 2016; Tong et al., 2023; Zhou et al., 2015). For example, the uptake of sulfasalazine increases in mice when co‐administered with 300–400 mg/kg curcumin (Kusuhara et al., 2012; Shukla et al., 2009). In this study, the plasma concentration–time curves of the cinnamic acid derivatives showed two peaks at 30 min and 3–6 h after the co‐administration of BGP and 200 mg/kg curcumin in the turmeric extract; these values were significantly higher than those obtained when BGP was administered alone (Figure 2b). As compounds with two peaks in the plasma concentration–time curves are considered to be the result of enterohepatic circulation (Yan et al., 2006), the first absorption phase after oral ingestion of the cinnamic acid derivatives and the intestinal reabsorption phase of the cinnamic acid derivatives excreted in the bile would increase because curcumin inhibits their transport to the intestine via BCRP. The elevated uptake of cinnamic acid derivatives in the body can enhance the pharmacological activities of BGP. For instance, when combined with herbal extracts, BGP is effective in improving cognitive function in elderly people, even at lower doses than when administered alone (Asama et al., 2020, 2021). In addition, artepillin C increases mitochondrial UCP1 expression and induces the formation of beige adipocytes in the white adipose tissue of mice (Nishikawa et al., 2020). Curcumin also enhances the induction of beige adipocyte formation by artepillin C (Nishikawa et al., 2019). Although the actual plasma artepillin C concentration was not confirmed in these studies, an increase in the plasma artepillin C concentration due to curcumin inhibition by BCRP may be one of the factors contributing to the synergistic effect.

BCRP expression is not restricted to the intestine but extends to the liver and kidney and is known to contribute to the urinary and biliary excretion of substrates (Mizuno & Sugiyama, 2002), leading to substrate accumulation in the body. In the present study, the plasma concentrations of artepillin C and drupanin did not differ at 24 h post‐ingestion between the rats administered BGP alone and those administered BGP and turmeric, suggesting that the effect of curcumin on the clearance of these compounds was less than that on their absorption (Figure 2b). This result could be related to the metabolism of curcumin in vivo. Curcumin is metabolized to glucuronide and sulfate conjugates, and the IC50 values of these conjugates for BCRP are higher than those of curcumin (Heger et al., 2013; Karibe et al., 2018). Although the amount of curcumin and its metabolites distributed in the liver and kidneys should be verified because of the dose of curcumin used, curcumin may not have reached these organs in sufficient amounts to inhibit BCRP.

In the bidirectional transport assay using MDCK II‐BCRP cells, the ER value of drupanin was 15.7, which was approximately 10‐fold higher than that of artepillin C (Table 3). These results suggested that the affinity of drupanin for BCRP was higher than that of artepillin C. However, the increases in the AUC values of artepillin C and drupanin in humans after co‐administration of BGP and the herbal extract mixture were 1.7‐ and 1.5‐fold, respectively, compared to that of BGP administration alone (Figure 1). Similarly, in rats, the AUC values of artepillin C and drupanin increased 1.4‐ and 1.3‐fold, respectively, owing to the co‐administration of BGP and turmeric extract (Figure 2a), suggesting no difference between the absorption of artepillin C and drupanin due to the co‐administration of BGP and turmeric extract in humans and rats. The difference between the in vitro and in vivo results suggests that the affinities of artepillin C and drupanin for BCRP are altered by metabolism in the gastrointestinal epithelium. Our previous studies have revealed that artepillin C and drupanin are absorbed into the body not only as aglycones but also as conjugation metabolites in humans and rats. Furthermore, artepillin C is mainly metabolized to glucuronide in both humans and rats, whereas drupanin metabolites are chiefly glucuronide in humans and sulfate in rats (Yamaga et al., 2021, 2022). Among the flavonoids, genistein has been reported to be excreted as a BCRP substrate following its metabolism to glucuronide or sulfate conjugates (Zhu et al., 2010). Ko‐143, a BCRP inhibitor, causes greater inhibition of the efflux of chrysin sulfate to the outside of cells than glucuronide, suggesting that chrysin sulfate has a higher affinity for BCRP than chrysin glucuronide (Ge et al., 2015). Therefore, the affinity of substrates for BCRP could be altered by conjugation metabolism; however, the pattern of changes in the binding affinity for BCRP is substrate‐dependent and not specifically defined. To understand the effect of BCRP on the absorption of artepillin C and drupanin, differences in the affinities of their conjugated metabolites for BCRP compared to aglycone had to be confirmed.

Generally, the absorption and metabolism of active constituents in foods and plants are influenced by the presence of other foods and herbal constituents (Colalto, 2010; Sun et al., 2019). However, the co‐administration of ginkgo leaf, coffee fruit, or soybean did not induce significant changes in the absorption of BGP components in rats (Figure 2a). In mice, the AUC and *C*_max_ of sulfasalazine were not altered by the co‐administration of a single dose of ginkgo leaves and sulfasalazine, whereas the oral administration of ginkgo leaf extract for 5 days significantly suppressed the intestinal BCRP expression levels and increased the AUC and *C*_max_ of sulfasalazine by twofold compared with the administration of ginkgo leaves alone (Kim et al., 2021). Similarly, the administration of 80 mg/kg Ginkgo leaf extract for 10 days was reported to increase the AUC and *C*_max_ of atorvastatin, a BCRP substrate, in rats (Ren et al., 2019). Therefore, repeated administration of a mixture of BGP and herbal extracts may further increase the absorption of cinnamic acid derivatives compared with a single administration. A mixture of flavonoids and soybean‐derived phosphatidylcholine was found to improve the absorption of flavonoids via physical treatment to form liposomes (Wang et al., 2015). Therefore, the absorption of cinnamic acid derivatives can be improved not only by co‐administration, as observed in the present study, but also by liposome formation with phospholipids. Contrarily, the AUC values of drupanin and 3,4‐dihydroxy‐5‐prenylcinnamic acid tended to increase with the co‐administration of gotu kola extract and BGP (Figure 2a, *p* = .08 and .053, respectively). Gotu kola contains asiaticoside, a saponin, as the characteristic component (Idris & Nadzir, 2021; Paine, 2020). Saponins, which are amphipathic compounds with surface activity (Kunjumon et al., 2022), may improve the membrane permeability of drupanin. The AUC of artepillin C was not changed by the co‐administration of gotu kola extract and BGP. Therefore, further detailed studies could shed light on the relationship between the differences in the structures and absorption processes of cinnamic acid derivatives.

## 5 CONCLUSION

To the best of our knowledge, this is the first study to demonstrate that the absorption of prenylated cinnamic acid derivatives, which are characteristic compounds of BGP, can be improved by curcumin‐mediated BCRP inhibition. The findings of this investigation provide valuable insights into various foods and herbal medicines containing cinnamic acid derivatives and prenylated compounds in relation to food–herb and herb–herb interactions in vivo.

## AUTHOR CONTRIBUTIONS

**Masayuki Yamaga:** Conceptualization (equal); data curation (equal); formal analysis (equal); investigation (equal); methodology (equal); writing – original draft (equal). **Hiroshi Kawabe:** Formal analysis (equal); investigation (equal). **Hiroko Tani:** Conceptualization (equal); data curation (equal); supervision (equal); validation (equal); writing – review and editing (equal). **Ayanori Yamaki:** Supervision (equal); validation (equal); writing – review and editing (equal).

## CONFLICT OF INTEREST STATEMENT

M.Y., H.K., H.T., and A.Y. are employees of Yamada Bee Company, Inc., a company engaged in research and commercial endeavors related to bee products. The authors declare no competing financial interests.

## ETHICAL STATEMENT

The protocol for this clinical trial was approved by Yamada Bee Co. Ethical Committee. The study was conducted in accordance with the ethical principles of the Declaration of Helsinki (approval no. 2020015). This animal study was approved by the Animal Research Ethics Committee of KAC Co., Ltd. (approval No. 22–1217). All rats were handled in accordance with the Animal Care and Use Regulations of KAC Co. Ltd.

## INFORMED CONSENT

All participants were informed of the research protocol and they have signed an informed consent form.

## Data Availability

The data underlying this study can be obtained from the corresponding author upon request.

## References

[fsn34116-bib-0001] Asama, T. , Hiraoka, T. , Ohkuma, A. , Okumura, N. , Yamaki, A. , Igase, M. , & Urakami, K. (2020). Cognitive improvement and safety assessment of a composite dietary supplement containing propolis extract, *Gingko biloba* extract, phosphatidylserine and curcumin in healthy mid‐ to senior age Japanese adults‐a placebo‐controlled, randomized, parallel‐group, double‐blind human clinical study. Japanese Pharmacology & Therapeutics, 48, 1805–1819.

[fsn34116-bib-0002] Asama, T. , Hiraoka, T. , Ohkuma, A. , Okumura, N. , Yamaki, A. , & Urakami, K. (2021). Cognitive improvement and safety assessment of a dietary supplement containing propolis extract in elderly Japanese: A placebo controlled, randomized, parallel group, double blind human clinical study. Evidence‐Based Complementary and Alternative Medicine, 2021, 6664217. 10.1155/2021/6664217 33680059 PMC7929669

[fsn34116-bib-0003] Bankova, V. S. , de Castro, S. L. , & Marcucci, M. C. (2000). Propolis: Recent advances in chemistry and plant origin. Apidologie, 31, 3–15. 10.1051/apido:2000102

[fsn34116-bib-0004] Bhargava, P. , Grover, A. , Nigam, M. , Kaul, A. , Doi, M. , Ishida, Y. , Kakuta, H. , Kaul, S. C. , Terao, K. , & Wadhwa, R. (2018). Anticancer activity of the supercritical extract of Brazilian green propolis and its active component, artepillin C: Bioinformatics and experimental analyses of its mechanisms of action. International Journal of Oncology, 52, 925–932. 10.3892/ijo.2018.4249 29393408

[fsn34116-bib-0005] Burdock, G. A. (1998). Review of the biological properties and toxicity of bee propolis (propolis). Food and Chemical Toxicology, 36, 347–363. 10.1016/s0278-6915(97)00145-2 9651052

[fsn34116-bib-0006] Cardoso, J. G. , Iorio, N. L. P. , Rodrigues, L. F. , Couri, M. L. B. , Farah, A. , Maia, L. C. , & Antonio, A. G. (2016). Influence of a Brazilian wild green propolis on the enamel mineral loss and *Streptococcus mutans*' count in dental biofilm. Archives of Oral Biology, 65, 77–81. 10.1016/j.archoralbio.2016.02.001 26871983

[fsn34116-bib-0007] Colalto, C. (2010). Herbal interactions on absorption of drugs: Mechanisms of action and clinical risk assessment. Pharmacology Research, 62, 207–227. 10.1016/j.phrs.2010.04.001 20399862

[fsn34116-bib-0008] de Albuquerque, N. C. P. , Tadini, M. C. , Forte, A. L. S. A. , Ballestero, G. , Teixeira, T. V. , de Oliveira, F. M. , Fagnon, M. S. , Jouaud, M. , Chantemargue, B. , Trouillas, P. , Berretta, A. A. , & Kerros, S. (2023). Citrus, milk thistle, and propolis extracts improved the intestinal permeability of curcuminoids from turmeric extract – An in silico and in vitro permeability Caco‐2 cells approach. ACS Food Science & Technology, 3, 113–122. 10.1021/acsfoodscitech.2c00312

[fsn34116-bib-0009] de Simone, R. , Ajmone‐Cat, M. A. , Tirassa, P. , & Minghetti, L. (2003). Apoptotic PC12 cells exposing phosphatidylserine promote the production of anti‐inflammatory and neuroprotective molecules by microglial cells. Journal of Neuropathology & Experimental Neurology, 62, 208–216. 10.1093/jnen/62.2.208 12578230

[fsn34116-bib-0010] Fiordalisi, S. A. L. , Honorato, L. A. , Loiko, M. R. , Avancini, C. A. M. , Veleirinho, M. B. R. , Filho, L. C. P. M. , & Kuhnen, S. (2016). The effects of Brazilian propolis on etiological agents of mastitis and the viability of bovine mammary gland explants. Journal of Dairy Science, 99, 2308–2318. 10.3168/jds.2015-9777 26723111

[fsn34116-bib-0011] Fitriana, L. A. , Anggadiredja, K. , Setiawan , & Adnyana, I. K. (2021). Twenty weeks of *Centella asiatica* improved cognitive function of women elderly with dementia. IOP Conference Series: Earth and Environmental Science, 755, 012064. 10.1088/1755-1315/755/1/012064

[fsn34116-bib-0012] Ge, S. , Gao, S. , Yin, T. , & Hu, M. (2015). Determination of pharmacokinetics of chrysin and its conjugates in wild‐type FVB and bcrp1 knockout mice using a validated LC‐MS/MS method. Journal of Agricultural and Food Chemistry, 63, 2902–2910. 10.1021/jf5056979 25715997

[fsn34116-bib-0013] Ge, S. , Yin, T. , Xu, B. , Gao, S. , & Hu, M. (2016). Curcumin affects phase II disposition of resveratrol through inhibiting efflux transporters MRP2 and BCRP. Pharmaceutical Research, 33, 590–602. 10.1007/s11095-015-1812-1 26502886 PMC4744546

[fsn34116-bib-0014] Ghisalberti, E. L. (1979). Propolis: A review. Bee World, 60, 59–84. 10.1080/0005772X.1979.11097738

[fsn34116-bib-0015] Heger, M. , van Golen, R. F. , Broekgaarden, M. , & Michel, M. C. (2013). The molecular basis for the pharmacokinetics and pharmacodynamics of curcumin and its metabolites in relation to cancer. Pharmacological Reviews, 66, 222–307. 10.1124/pr.110.004044 24368738

[fsn34116-bib-0016] Huang, S. M. , Zhang, L. , & Giacomini, K. M. (2010). The international transporter consortium: A collaborative group of scientists from academia, industry, and the FDA. Clinical Pharmacology & Therapeutics, 87, 32–36. 10.1038/clpt.2009.236 20019700

[fsn34116-bib-0017] Idris, F. N. , & Nadzir, M. M. (2021). Comparative studies on different extraction methods of *Centella asiatica* and extracts bioactive compounds effects on antimicrobial activities. Antibiotics, 10, 457. 10.3390/antibiotics10040457 33920563 PMC8073564

[fsn34116-bib-0018] Kano, Y. , Horie, N. , Doi, S. , Aramaki, F. , Maeda, H. , Hiragami, F. , Kawamura, K. , Motoda, H. , Koike, Y. , Akiyama, J. , Eguchi, S. , & Hashimoto, K. (2008). Artepillin C derived from propolis induces neurite outgrowth in PC12m3 cells via ERK and p38 MAPK pathways. Neurochemical Research, 33, 1795–1803. 10.1007/s11064-008-9633-9 18338254

[fsn34116-bib-0019] Karibe, T. , Imaoka, T. , Abe, K. , & Ando, O. (2018). Curcumin as an *in vivo* selective intestinal breast cancer resistance protein inhibitor in cynomolgus monkeys. Drug Metabolism and Disposition, 46, 667–679. 10.1124/dmd.117.078931 29358184

[fsn34116-bib-0020] Kim, J. K. , Choi, M. S. , Kim, J. Y. , Yu, J. S. , Seo, J. I. , Yoo, H. H. , & Kim, D. H. (2021). *Ginkgo biloba* leaf extract suppresses intestinal human breast cancer resistance protein expression in mice: Correlation with gut microbiota. Biomedicine and Pharmacotherapy, 140, 111712. 10.1016/j.biopha.2021.111712 34010745

[fsn34116-bib-0021] Koya‐Miyata, S. , Arai, N. , Mizote, A. , Taniguchi, Y. , Ushio, S. , Iwaki, K. , & Fukuda, S. (2009). Propolis prevents diet‐induced hyperlipidemia and mitigates weight gain in diet‐induced obesity in mice. Biological and Pharmaceutical Bulletin, 32, 2022–2028. 10.1248/bpb.32.2022 19952422

[fsn34116-bib-0022] Kunjumon, R. , Johnson, A. J. , & Boby, S. (2022). *Centella asiatica*: Secondary metabolites, biological activities and biomass sources. Phytomedicine Plus, 2, 100176. 10.1016/j.phyplu.2021.100176

[fsn34116-bib-0023] Kusuhara, H. , Furuie, H. , Inano, A. , Sunagawa, A. , Yamada, S. , Wu, C. , Fukizawa, S. , Morimoto, N. , Ieiri, I. , Morishita, M. , Sumita, K. , Mayahara, H. , Fujita, T. , Maeda, K. , & Sugiyama, Y. (2012). Pharmacokinetic interaction study of sulphasalazine in healthy subjects and the impact of curcumin as an *in vivo* inhibitor of BCRP. British Journal of Pharmacology, 166, 1793–1803. 10.1111/j.1476-5381.2012.01887.x 22300367 PMC3402804

[fsn34116-bib-0024] Lee, C. K. , Ki, S. H. , & Choi, J. S. (2011). Effects of oral curcumin on the pharmacokinetics of intravenous and oral etoposide in rats: Possible role of intestinal CYP3A and P‐gp inhibition by curcumin. Biopharmaceutics & Drug Disposition, 32, 245–251. 10.1002/bdd.754 21506134

[fsn34116-bib-0025] Liu, G. Z. , Niu, T. T. , Yu, Q. , Xu, B. L. , Li, X. Q. , Yuan, G. B. , Yang, T. T. , Li, H. Q. , & Sun, Y. (2023). Ginkgolide attenuates memory impairment and neuroinflammation by suppressing the NLRP3/caspase‐1 pathway in Alzheimer's disease. Aging, 15, 10237–10252. 10.18632/aging.205072 37793010 PMC10599747

[fsn34116-bib-0026] Mizuno, N. , & Sugiyama, Y. (2002). Drug transporters: Their role and importance in the selection and development of new drugs. Drug Metabolism and Pharmacokinetics, 17, 93–108. 10.2133/dmpk.17.93 15618657

[fsn34116-bib-0027] Nishikawa, S. , Hydo, T. , Aoyama, H. , Miyata, R. , Kumazawa, S. , & Tsuda, T. (2020). Artepillin C, a key component of Brazilian propolis, induces thermogenesis in inguinal white adipose tissue of mice through a creatine‐metabolism‐related thermogenic pathway. Journal of Agricultural and Food Chemistry, 68, 1007–1014. 10.1021/acs.jafc.9b07080 31914311

[fsn34116-bib-0028] Nishikawa, S. , Kamiya, M. , Aoyama, H. , Yoshimura, K. , Miyata, R. , Kumazawa, S. , & Tsuda, T. (2019). Co‐administration of curcumin and artepillin C induces development of brown‐like adipocytes in association with local norepinephrine production by alternatively activated macrophages in mice. Journal of Nutritional Science and Vitaminology, 65, 328–334. 10.3177/jnsv.65.328 31474682

[fsn34116-bib-0029] Paine, M. F. (2020). Natural products: Experimental approaches to elucidate disposition mechanisms and predict pharmacokinetic drug interactions. Drug Metabolism and Disposition, 48, 956–962. 10.1124/dmd.120.000182 32816868 PMC7543467

[fsn34116-bib-0030] Park, Y. K. , Alencar, S. M. , & Aguiar, C. L. (2002). Botanical origin and chemical composition of Brazilian propolis. Journal of Agricultural and Food Chemistry, 50, 2502–2506. 10.1021/jf011432b 11958612

[fsn34116-bib-0031] Phan, M. A. T. , Paterson, J. , Bucknall, M. , & Arcot, J. (2018). Interactions between phytochemicals from fruits and vegetables: Effects on bioactivities and bioavailability. Critical Reviews in Food Science and Nutrition, 58, 1310–1329. 10.1080/10408398.2016.1254595 27880063

[fsn34116-bib-0032] Ren, Y. , Li, H. , & Liu, X. (2019). Effects of *ginkgo* leaf tablets on the pharmacokinetics of atovastatin in rats. Pharmaceutical Biology, 57, 403–406. 10.1080/13880209.2019.1622569 31188698 PMC6566491

[fsn34116-bib-0033] Scholz, S. , & Williamson, G. (2007). Interactions affecting the bioavailability of dietary polyphenols *in vivo* . International Journal for Vitamin and Nutrition Research, 77, 224–235. 10.1024/0300-9831.77.3.224 18214024

[fsn34116-bib-0034] Shukla, S. , Zaher, H. , Hartz, A. , Bauer, B. , Ware, J. A. , & Ambudkar, S. V. (2009). Curcumin inhibits the activity of ABCG2/BCRP1, a multidrug resistance‐linked ABC drug transporter in mice. Pharmaceutical Research, 26, 480–487. 10.1007/s11095-008-9735-8 18841445 PMC2662629

[fsn34116-bib-0035] Silveira, M. A. D. , Jong, D. D. , Berretta, A. A. , Galvão, E. B. D. S. , Ribeiro, J. C. , Cerqueira‐Silva, T. , Amorim, T. C. , da Conceição, L. F. M. R. , Gomes, M. M. D. , Teixeira, M. B. , de Souza, S. P. , Santos, M. H. C. A. D. , Martin, R. L. A. S. , Silva, M. O. , Lírio, M. , Moreno, L. , Sampaio, J. C. M. , Mendonça, R. , Ultchak, S. S. , … BeeCovid Team . (2021). Efficacy of Brazilian green propolis (EPP‐AF®) as an adjunct treatment for hospitalized COVID‐19 patients: A randomized, controlled clinical trial. Biomedicine & Pharmacotherapy, 138, 111526. 10.1016/j.biopha.2021.111526 34311528 PMC7980186

[fsn34116-bib-0036] Sun, S. , Wang, Y. , Wu, A. , Ding, Z. , & Liu, X. (2019). Influence factors of the pharmacokinetics of herbal resourced compounds in clinical practice. Evidence‐Based Complementary and Alternative Medicine, 2019, 1983780. 10.1155/2019/1983780 30949215 PMC6425497

[fsn34116-bib-0037] Takahashi, H. , Nguyen, B. C. Q. , Uto, Y. , Shahinozzaman, M. , Tawata, S. , & Maruta, H. (2017). 1,2,3‐triazolyl esterization of PAK1‐blocking propolis ingredients, artepillin C (ARC) and caffeic acid (CA), for boosting their anti‐cancer/anti‐PAK1 activities along with cell‐permeability. Drug Discoveries & Therapeutics, 11, 104–109. 10.5582/ddt.2017.01009 28442677

[fsn34116-bib-0038] Tani, H. , Hasumi, K. , Tatefuji, T. , Hashimoto, K. , Koshino, H. , & Takahashi, S. (2010). Inhibitory activity of Brazilian green propolis components and their derivatives on the release of cys‐leukotrienes. Bioorganic & Medicinal Chemistry, 18, 151–157. 10.1016/j.bmc.2009.11.007 19942440

[fsn34116-bib-0039] Tani, H. , Hikami, S. , Takahashi, S. , Kimura, Y. , Matsuura, N. , Nakamura, T. , Yamaga, M. , & Koshino, H. (2019). Isolation, identification, and synthesis of a new prenylated cinnamic acid derivative from Brazilian green propolis and simultaneous quantification of bioactive components by LC‐MS/MS. Journal of Agricultural and Food Chemistry, 67, 12303–12312. 10.1021/acs.jafc.9b04835 31597041

[fsn34116-bib-0040] Tani, H. , Yamaga, M. , Sekiya, T. , Isohama, Y. , Koshino, H. , Nogawa, T. , Yamaki, A. , & Takahashi, S. (2023). Identification of a new pyrrolyl pyridoindole alkaloid, melpyrrole, and flazin from honey and their cough‐suppressing effect in Guinea pigs. Journal of Agricultural and Food Chemistry, 71, 13805–13813. 10.1021/acs.jafc.3c03864 37683090

[fsn34116-bib-0041] Tong, L. , Zhou, Z. , Wang, G. , & Wu, C. (2023). A self‐microemulsion enhances oral absorption of docetaxel by inhibiting P‐glycoprotein and CYP metabolism. Drug Delivery and Translational Research, 13, 983–993. 10.1007/s13346-022-01255-x 36515864

[fsn34116-bib-0042] Toreti, V. C. , Sato, H. H. , Pastore, G. M. , & Park, Y. K. (2013). Recent progress of propolis for its biological and chemical compositions and its botanical origin. Evidence‐Based Complementary and Alternative Medicine, 2013, 697390. 10.1155/2013/697390 23737843 PMC3657397

[fsn34116-bib-0043] Wang, H. , Cui, Y. , Fu, Q. , Deng, B. , Li, G. , Yang, J. , Wu, T. , & Xie, Y. (2015). A phospholipid complex to improve the oral bioavailability of flavonoids. Drug Development and Industrial Pharmacy, 41, 1693–1703. 10.3109/03639045.2014.991402 25496311

[fsn34116-bib-0044] Wu, Z. , Zhu, A. , Takayama, F. , Okada, R. , Liu, Y. , Harada, Y. , Wu, S. , & Nakanishi, H. (2013). Brazilian green propolis suppresses the hypoxia‐induced neuroinflammatory responses by inhibiting NF‐κB activation in microglia. Oxidative Medicine and Cellular Longevity, 2013, 906726. 10.1155/2013/906726 23983903 PMC3747398

[fsn34116-bib-0045] Xiong, S. , Su, X. , Kang, Y. , Si, J. , Wang, L. , Li, X. , & Ma, K. (2023). Effect and mechanism of chlorogenic acid on cognitive dysfunction in mice by lipopolysaccharide‐induced neuroinflammation. Frontiers in Immunology, 14, 1178188. 10.3389/fimmu.2023.1178188 37292216 PMC10244504

[fsn34116-bib-0046] Yamaga, M. , Tani, H. , & Kaeko, M. (2022). Metabolic pathways of cinnamic acid derivatives in Brazilian green propolis in rats. Bioscience, Biotechnology, and Biochemistry, 86, 1075–1084. 10.1093/bbb/zbac076 35612978

[fsn34116-bib-0047] Yamaga, M. , Tani, H. , Nishikawa, M. , Fukaya, K. , Ikushiro, S. , & Kaeko, M. (2021). Pharmacokinetics and metabolism of cinnamic acid derivatives and flavonoids after oral administration of Brazilian green propolis in humans. Food & Function, 12, 2520–2530. 10.1039/d0fo02541k 33688872

[fsn34116-bib-0048] Yan, M. , Li, P. , Chen, D. , Fang, T. , Li, H. , & Su, W. (2006). LC/MS/MS quantitation assay for pharmacokinetics of naringenin and double peaks phenomenon in rats plasma. International Journal of Pharmaceutics, 307, 292–299. 10.1016/j.ijpharm.2005.10.018 16289985

[fsn34116-bib-0049] Yu, Y. , Shen, Q. , Lai, Y. , Park, S. Y. , Ou, X. , Lin, D. , Jin, M. , & Zhang, W. (2018). Anti‐inflammatory effects of curcumin in microglia cells. Frontiers in Pharmacology, 9, 386. 10.3389/fphar.2018.00386 29731715 PMC5922181

[fsn34116-bib-0050] Zhou, Q. , Ye, M. , Zhang, H. , Chen, Q. , Huang, S. , & Su, S. (2015). Curcumin improves the tumoricidal effect of mitomycin C by suppressing ABCG2 expression in stem cell‐like breast cancer cells. PLoS One, 10, e0136694. 10.1371/journal.pone.0136694 26305906 PMC4549178

[fsn34116-bib-0051] Zhu, A. , Wu, Z. , Zhong, X. , Ni, J. , Li, Y. , Meng, J. , Du, C. , Zhao, X. , Nakanishi, H. , & Wu, S. (2018). Brazilian green propolis prevents cognitive decline into mild cognitive impairment in elderly people living at high altitude. Journal of Alzheimer's Disease, 63, 551–560. 10.3233/jad-170630 29630549

[fsn34116-bib-0052] Zhu, W. , Xu, H. , Wang, S. W. J. , & Hu, M. (2010). Breast cancer resistance protein (BCRP) and sulfotransferases contribute significantly to the disposition of genistein in mouse intestine. The AAPS Journal, 12, 525–536. 10.1208/s12248-010-9209-x 20582579 PMC2976988

[fsn34116-bib-0053] Zou, L. , Pottel, J. , Khuri, N. , Ngo, H. X. , Ni, Z. , Tsakalozou, E. , Warren, M. S. , Huang, Y. , Shoichet, B. K. , & Giacomini, K. M. (2020). Interactions of oral molecular excipients with breast cancer resistance protein, BCRP. Molecular Pharmaceutics, 17, 748–756. 10.1021/acs.molpharmaceut.9b00658 31990564 PMC8177814

